# Editorial: Multidisciplinary Approach to the Diagnosis and Therapy of Skin Neoplasms

**DOI:** 10.3389/fonc.2022.939200

**Published:** 2022-07-14

**Authors:** Andrea Ronchi, Giuseppe Argenziano, Gabriella Brancaccio, Aimilios Lallas, Renato Franco

**Affiliations:** ^1^ Pathology Unit, Department of Mental and Physical Health and Preventive Medicine, University of Campania “Luigi Vanvitelli”, Naples, Italy; ^2^ Dermatology Unit, Department of Mental and Physical Health and Preventive Medicine, University of Campania “Luigi Vanvitelli”, Naples, Italy; ^3^ First Department of Dermatology, School of Medicine, Aristotle University, Thessaloniki, Greece

**Keywords:** skin cancer, dermoscopy, melanoma, diagnosis, confocal microscopy

Skin cancer is an extremely heterogenous group of neoplasms, including tumors of the epidermis (basal cell carcinoma, squamous cell carcinoma), tumors of the dermal adnexa (adnexal carcinomas), tumors of melanocytes (cutaneous melanoma), tumors of soft tissue (angiosarcoma and other sarcomas) and tumors of neuroendocrine cells (Merkel cell carcinoma). Despite the better knowledge of its pathological and molecular features, actually skin cancer is a clinical challenge with relevant consequences in terms of morbidity and mortality. Indeed, skin cancer, including cutaneous melanoma and non-melanoma skin cancer, is the most common cancer worldwide. It has been calculated that 1 in 5 subjects will develop skin cancer by the age of 70 in the U.S., and more than 2 subjects die of skin cancer in the U.S. every hour (1, 2). Co-morbidities of patients affected by skin cancer, the challenges of the histological diagnosis, the development of new technologies for the *in-vivo* screening of the patients and the early diagnosis, make the multidisciplinary approach mandatory. In this setting, the aim of this Research Topic is to address the main topics about skin cancer from a multidisciplinary point of view. Cutaneous melanoma is certainly a hot issue, and this Research Topic includes articles about new classification, histological diagnosis, *in-vivo* technologies, new therapies. Umano et al., evaluated the value of Preferentially expressed Antigen in Melanoma (PRAME) for differential diagnosis of spitzoid melanocytic lesions in pediatric patients. Pediatric melanoma is also the topic of a retrospective study by Ryan et al., which provided clinical and prognostic information about this rare neoplasm in a challenging patient group. Ferrara and Argenziano., revised the WHO 2018 classification of cutaneous melanocytic neoplasms, with particular emphasis on intermediate melanocytic tumors and immunohistochemical and molecular algorithms needed to address the morphological ambiguous cases to a correct clinical management.

In the last decades, the development of new technologies has dramatically changed the diagnosis of skin cancer, mainly cutaneous melanoma, and it is to be expected that technologies will acquire an ever-greater role in the next future (3). In the review by Belfiore et al., the Authors examine the role of High frequency Ultrasound (HFUS) in the diagnosis of skin cancer, while Broggi et al., focused on the correlation between *in vivo* reflectance confocal microscopy and horizontal histopathology.

With regards to the management, immunotherapy is certainly one of the most important novelty in the therapy of skin cancer in the last years, but the effectiveness of immunotherapy in advanced melanoma cannot be predicted (4). In the bibliometric analysis by Zhang et al., immunotherapy is confirmed as a hot issue in cutaneous melanoma research. Palmieri et al., examine the potential role of molecular alterations linked to genetic instability in cutaneous melanoma as predictive biomarkers for response to immunotherapy. Zheng et al. examine current evidence about the effectiveness of immune checkpoint inhibitors therapy in advanced acral melanoma. Melanoma metastases of unknown primary is a relatively common clinical challenge and the recent improvements about clinical management, therapy and prognostic factors are evaluated by the retrospective study by Del Fiore et al.

Although most non-melanoma skin cancer is easily healed with surgery alone, some neoplasms are extremely relevant in terms of morbidity and mortality. This Research Topic includes several articles about the therapy and the prognostic evaluation of these neoplasms. Pampena et al., define the clinical-dermoscopic findings of aggressive subtypes of basal cell carcinoma, while Russo et al., provide evidence that the expression of Carbonic Anhydrase IX may be used as a prognostic marker in basal cell carcinoma and has a potential therapeutic role for target therapy in advanced cases. Data about the use of sonidegib for the treatment of advanced basal cell carcinoma are detailed by Brancaccio et al. Hashimoto et al., analyze the impact of mucosal involvement and surgical treatment on the survival of patients with extramammary Paget’s disease. Bi et al., examine the different therapeutic chances for cutaneous angiosarcoma, while Feng et al., describe a case of recurrent Merkel cell carcinoma responding to Tyrosine Kinase Inhibitor Apatinib.

Lastly, the general clinical context always plays an important role in the management of patients affected by skin cancer. A possible misinterpretation of PET/CT images caused by the recent COVID-19 vaccination is described in the case report by Czepczynski et al., Venanzi Rullo et al., describe the peculiar findings of non-melanoma skin cancer in patients affected by HIV. Immunodepression influences not only the epidemiology and the biology of skin cancer, but mainly the clinical management of the patients. Interestingly, Crisafulli et al. systematically review the cutaneous malignancy risk in patients treated for chronic inflammatory cutaneous diseases.

Altogether, the different contributions to this Research Topic offer a comprehensive analysis of improvements in diagnosis and therapy of skin cancer, highlighting the importance of a multidisciplinary approach to this heterogeneous group of neoplasms.

## Author Contributions

All authors equally contributed to the manuscript. All authors read and approved the final manuscript.

## Conflict of Interest

The authors declare that the research was conducted in the absence of any commercial or financial relationships that could be construed as a potential conflict of interest.

## Publisher’s Note

All claims expressed in this article are solely those of the authors and do not necessarily represent those of their affiliated organizations, or those of the publisher, the editors and the reviewers. Any product that may be evaluated in this article, or claim that may be made by its manufacturer, is not guaranteed or endorsed by the publisher.
